# The Medical Treatment of Perityphilitis and Appendicitis

**Published:** 1892-08-20

**Authors:** 


### THE MEDICAL TREATMENT OF PERITYPHLITIS AND APPENDICITIS.

Some physicians must feel almost ashamed to treat cases of appendicitis instead of handing them over to their surgical confrères, for during the past twelve months medical literature has been flooded with reports of cases successfully treated by abdominal section. True it is that not so long ago the number of patients who were allowed to die for want of prompt surgical help must have been appalling, and we cannot wonder then that, as the results of abdominal surgery in this disease are so brilliant in comparison with the past state of affairs, we are going too far in the other direction and forgetting that many cases are better treated by physicians in the light of recent knowledge.

In a most interesting and instructive article on this subject, Dr. W. H. Link* (Petersburg, Ind.) remarks that the first and greatest advance made in this disease was in a better knowledge of its pathology. So long as typhlitis and perityphlitis were the nearest approach to a correct understanding of the condition, just so long was treatment uncertain and shadowy. We now know that a vast majority of the inflammatory troubles located in the ileo-cæcal region are, primarily, due to inflammation, ulceration, or perforation of the appendix vermiformis. We have learned that the question of therapy or surgery depends upon the advance made in the general progress of the case from a simple catarrhal or adhesive inflammation to a gangrenous perforation. Unfortunately we are not yet far enough advanced in our diagnostic resources to determine with positive certainty the exact status of a case from the rational symptoms or physical signs. There are no pathognomonic symptoms, no invariable or infallible physical reactions or conditions that, like guide boards, point both forward and backward, marking the exact distance alike from the inception to the end. But while both diagnosis and prognosis are only a balancing of probabilities, the indications for a given line of treatment, founded either on an enlightened experience or on scientific research, are unequivocal. Locking up of the bowels with opium is supreme folly, for they are already locked in a vast majority of cases by the paralysis of distension and the obtunded sensibility induced by disease. But opium does not only lock up the bowels. The secretions of the entire alimentary canal are deranged, and some of the emunctories in other parts of the system fail in their activities under the influence of this baleful drug. The author's experience leads him to think that most frequently the condition under consideration is due to traumatisms from without or from within, as when set up by foreign bodies, hardened fæces, improper food, or the invasion of pyogenic bacteria. In such conditions, says Dr. Link, the indications are plain: To get rid of the irritating material by placing the absorbing and digesting powers of the peritoneum at the best advantage; to lessen inflammation by depleting the local engorgement; to sweep away bacteria and their poisonous ptomaines in a copious exudation of fluid from the capillaries of the bowel. Now, instead of giving opium and applying poultices, the indications are far better met by a full dose of the sulphate of magnesium every hour till free watery discharges occur. Then absolute rest in bed and a strictly fluid diet. Treatment on these lines proved highly successful in Dr. Link's hands. His concluding remarks are worthy of careful note: "In the commencement of an attack give salines often and liberally, till the gut is completely emptied. Advise perfect rest in bed. Forbid any but liquid nourishment. If pain is severe, apply counter irritation and dry heat locally till salines act. If the patient improves, wait. If the pulse grows worse, if the temperature rises, if pain increases, if tumefaction becomes larger, if tenderness becomes more marked, operate. At no time give morphine, but consider an increase of pain sufficient to demand relief by opium as imperative, unequivocal, and emphatic indications for surgical interference."

* *New York Medical Journal*, January 9th, 1892.

The inadvisability of too early resort to surgical interference is emphasised by Dr. Saundby's recent record of fifteen cases of perityphlitis. Of these, only one was subjected to operation, and this was the only fatal case. A large majority were males, and in six there was a tuberculous history. The duration varied much, but in several instances a cure resulted in less than three weeks. The treatment adopted was very similar to that of Dr. Link, viz., free evacuation of the bowels and hot fomentations or the ice bag, with the addition, in chronic cases, of repeated blisterings over the tumour. Dr. Saundby used mostly calomel, hot seidlitz powders, and enemata. This authority does not believe it possible to distinguish between cases in which the appendix is actually the seat of inflammation and those in which it is not, and considers that such a distinction is of no practical moment.

Now it is obvious to every observing practitioner that these milder cases are most suitably treated by the means we have indicated, but our difficulty is to determine what are the "milder cases," and when, medical treatment having failed, to call in a surgeon. One thing is certain, that precious time must not be lost. If a case is not going to yield without an operation, the sooner it is performed the better chance has the patient. What precious time is so often wasted in vain attempts to reduce a strangulated hernia! We can lay down a definite rule in the case of hernia, and say that if called early in the case we may make one thorough, but careful, gentle, and skilful attempt to reduce under an anæsthetic, and then, if unsuccessful, operate, if possible, at once. Let the same principle guide us with regard to appendicitis. Of course, if there be any indication that the appendix is gangrenous or an abscess has formed, nothing but an immediate operation is admissible, but the same rule applies to cases of strangulated hernia.

Some very useful hints, based on practical experience, are offered by Mr. Thomas Jones,* of Manchester. He recommends that operation is necessary if by the second (certainly by the third) day there is marked abdominal pain in the right iliac fossa, especially if tenderness exists at

* *Med. Chron.*, p. 217.

McBurney's point (two inches from the right anterior superior iliac spine in a line extending from this process to the umbilicus), attended possibly with nausea and vomiting; and, further, if there is rigidity of the abdominal wall. As the cases vary so much in severity, no absolute rule can be laid down; we should, in fact, be guided by the symptoms. How long should the physician go on with the medical treatment of cases of appendicitis? (1) In acute perforative appendicitis, ushered in by sudden severe pain in the cæcal region, especially if accompanied by acute peritonitis, an operation should be resorted to without any delay, and also in recurrent appendicitis with elevation of temperature. (2) We must operate in cases where the fever is persistently high (100 deg. to 102 deg.) and refuses to yield to medical treatment. (3) A symptom which points to the necessity for operation is œdema of the abdominal wall; although rarely present, it invariably indicates the presence of pus.

Mr. Jones is of opinion that operative measures should be adopted at a much earlier period than has been heretofore usual, these consist in laying open the abscess cavity, washing it out, and introducing a drain.

